# Statistical validation of a new helical tomotherapy patient transfer station

**DOI:** 10.1120/jacmp.v10i3.3060

**Published:** 2009-06-02

**Authors:** Audrey H. Zhuang, An Liu, Timothy E. Schultheiss, Jeffrey Y.C. Wong

**Affiliations:** ^1^ Department of Radiation Oncology City of Hope Cancer Center Duarte California USA

**Keywords:** TomoTherapy, patient transfer, treatment planning

## Abstract

The purpose of this work is to evaluate statistically the accuracy of a patient transfer station (PTS), which automatically converts one planning‐station‐generated treatment plan to another one with a different beam model. In our department we have installed two Hi•Art tomotherapy systems (TomoTherapy Inc., Madison, WI), and patients often need to be transferred from one tomotherapy unit to the other. Thirty patients who underwent patient transfer between the two systems were evaluated. For each patient, dose differences between his/her original plan and PTS‐transferred plan were evaluated by comparing doses at 10 randomly selected positions in his/her CT images. The Pearson indexes were calculated to analyze the relationship of the deviations to other parameters, which include absolute dose levels, sites (targets or normal tissues), and the dose accuracy of original plans and that of transferred plans. The dose accuracy of a treatment plan was determined by comparing delivered doses at the center of a 30cm×30cm×12cm solid water phantom to planned doses at the same position. The calculated dose difference between original and transferred plans was, on average, 0.8%±0.5%; the maximum deviation in absolute values was 1.9% in target volumes and 2.5% in normal organs. The errors generated during PTS‐based transferring process were random and did not show correlation with other parameters. The PTS took less than 10 minutes to generate a backup plan, much less than the approximately two hours needed to create a duplicate plan manually. The results show that a PTS‐transferred plan is an acceptable match to the original plan. With a physician's approval, a transferred plan is acceptable for treatment without the necessity of being revalidated in phantom. Thus far, all of our PTS plans have been approved by the treating physician without further optimization.

PACS number: 87.55.km, 87.55.D‐

## I. INTRODUCTION

Helical tomotherapy provides highly conformal and accurate dose delivery by combining the ability of intensity modulated radiation therapy and an image‐guided patient setup verification subsystem.^(^ ^1^ ^,^ ^2^ ^)^ In our department we have installed two non‐twinned Hi•Art tomotherapy systems, and in this paper they are referred to as Tomo1 and Tomo2. When any unanticipated functional failure occurs on one of the units, patients would be transferred to the other unit to receive backup treatments. To facilitate patient transfer between tomotherapy systems, a new software module called the Patient Transfer System (PTS), was developed by TomoTherapy Inc (Madison, WI). Non‐twinned tomotherapy systems are different in source output (i.e. dose rate), beam energy, and jaw settings (i.e. jaw sizes). The PTS automatically scales a sinogram, changes individual leaf open times, and adjusts the pitch to allow a transferred plan to dosimetrically match an original plan. First, the PTS adjusts the pitch based on jaw size differences. The pitch is defined as the ratio of the couch travel distance for a complete gantry rotation divided by slice width at the axis of rotation, and the slice width is determined by the jaw size. The pitch is adjusted so that the product of the pitch and the jaw size remains constant. Second, the PTS scales a sinogram to scale leaf open times, so that the differences in output (dose delivered per minute) and jaw sizes are compensated. Third, the PTS modifies a sinogram to correct the differences in MLC properties such as leaf fluence output factors and leaf latency values.

**Figure 1:**
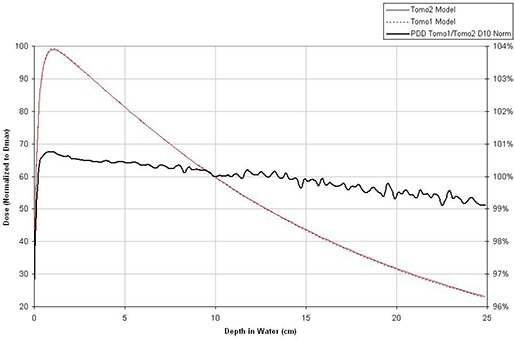
Percent depth dose (PDD) of Tomo1 and Tomo2 beam models.

The new PTS algorithm, on the other hand, does not correct differences in beam energy and beam profile shapes between tomotherapy systems. Between our non‐twinned machines there is a small difference in beam quality. As shown in Fig. 1, one machine's beam attenuates slightly differently in water than the other machine, which can cause dose differences between calculated doses when using the same leaf delivery sinogram. Secondly, although the PTS adjusts the pitch to compensate for the jaw size differences, the helical junctioning effect presented as a longitudinal “ripple” in patient's dose volume may still cause dose differences. Thirdly, a cutoff threshold is applied and small leaf open times that are under the threshold are closed. Due to the thresholding characteristics, very small sinogram differences induced during patient transfer may cause different number and pattern of leaves under the cutoff threshold and magnify sinogram differences. The uncertainty introduced by each factor is difficult to predict; therefore in this report, we will apply the PTS to transfer a large number of clinical treatment plans and evaluate the dose deviations between transferred plans and original plans statistically.

## II. MATERIALS AND METHODS

### A. the patient transfer station

**Figure 2:**
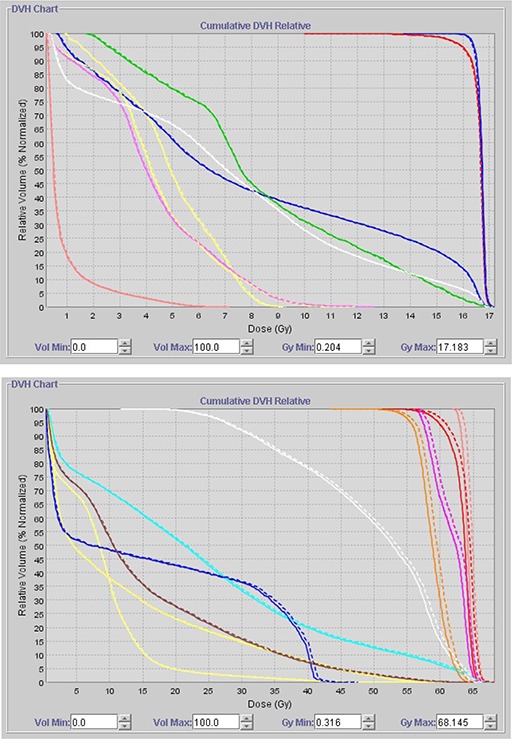
Patient transfer process from Tomo1 to Tomo2. The PTS stands for patient transfer station; OS stands for operating station; DS stands for database station; and PS stands for planning station.

Two Hi•Art Patient Transfer Stations (PTS) Version 2.2.2 have been installed in our department, each connecting to one of our two tomotherapy systems. Each PTS takes a plan archived from an original tomotherapy system (e.g. Tomo1) and changes it into another plan that can be installed and delivered on the other system (e.g. Tomo2). Figure 2 illustrates the flowchart of the patient transfer process.

### B. Patients

Thirty patients who underwent patient transfer were investigated. Disease sites include the prostate, bone marrow, head and neck, pancreas, and brain. We first created treatment plans using either one of our tomotherapy systems, then transferred the plans to the other system using the PTS. Each transferred plan was approved by an attending physician before being used for treatment.

### C. Dose comparison

Although tomotherapy treatment planning software generates dosimetrical statistics (i.e., max dose, min dose, mean dose, etc.) after the calculation of a plan's final dose, the statistics would not be performed for a transferred plan that was retrieved by the planning station. In order to quantitatively investigate dose differences between a transferred and an original plan, we obtained and compared absolute doses at randomly selected positions on patients’ images. Five positions were selected in a target volume and another five in a region at risk (RAR) using the dose investigation tool in the PTS. If multiple RARs exist in a patient's images, we selected the closest one to the target volume (e.g. the bladder or rectum in a prostate case, the kidney in a pancreas case, and the eye in a brain case). Those RARs are typically located in high‐dose gradient areas where the greatest dose deviations between transferred plans and original plans would be likely to occur.

### D. Delivery quality assurance

Verification of dose delivery of a transferred plan, referred to as delivery quality assurance (DQA), was done before it is used for treatment. DQA is done by delivering the treatment plan to a 30cm×30cm×12cm MedTec solid water phantom. We measured an absolute dose deposited at the center of the phantom using a 0.6 cm^3^ PTW Farmer ion chamber. The isodose distribution was obtained at the same time using an EDR2 film that was placed in a coronal plane of the phantom at the depth of 5 cm. The dose differences between a plan and its DQA measurement demonstrate the uncertainties of a treatment plan delivery.

### E. Correlation analyses

In order to determine the relationships between patient transfer‐induced dose uncertainties and other dose uncertainties in the planning process, we analyzed their correlations using the Pearson product‐moment correlation coefficient, which reflects a linear relationship between two data sets and is mathematically described in Eq. (1), ^(3)^

(1) $$r=\frac{\sum (X-\bar{X})(Y-\bar{Y})}{\sqrt{\sum {\left(X-\bar{X}\right)}^{2}\sum {\left(Y-\bar{Y}\right)}^{2}}}$$

where *r* is the Pearson product‐moment correlation coefficient ranging from −1.0 to 1.0; *X* and *Y* represent two data set arrays, and $\bar{X}$ and $\bar{Y}$ represent the sample means of data set *X* and *Y* respectively. We analyzed the relationship of the deviations induced during patient transfer to original plan delivery uncertainties and to transferred plan delivery uncertainties, respectively.

## III. RESULTS

### A. DVH comparison

**Figure 3:**
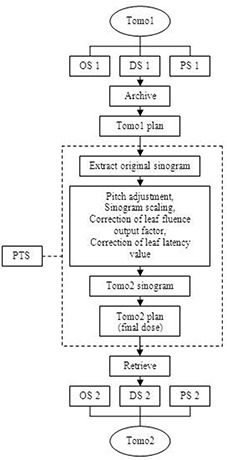
Illustration of DVH composite. The DVH of an original plan is represented in solid lines and that of a transferred plan in dashed lines; (a) illustrates one of the best comparisons of a transferred plan to an original plan; (b) illustrates one of the worst comparisons of a transferred plan to an original plan.

Figure 3 illustrates a composite of dose volume histograms (DVHs) for an original and a transferred plan. The average dose differences in absolute values for the right‐most DVH lines shown in Fig. 3(a) and 3(b) are 0.5% and 1.5%, respectively.

### B. Statistics of patient transfer uncertainties and their relationship to other uncertainties

**Figure 4:**
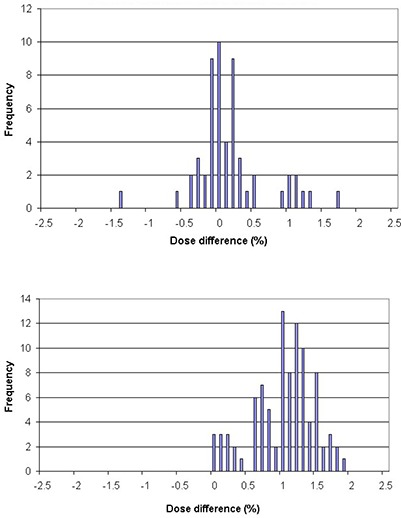
Frequency of target dose deviations in: (a) plans transferred from Tomo1 to Tomo2; and (b) plans transferred from Tomo2 to Tomo1.

The calculated dose difference between original and transferred plans was, on average, 0.8%±0.5%; the maximum deviation in absolute values was 1.9% in target volumes. The analyses of dose comparison show that the plans transferred from Tomo1 to Tomo2 present slightly different dose deviations than the plans transferred from Tomo2 to Tomo1. The former has an average dose deviation of 0.1%±0.5% and the latter has an average deviation of 1.0%±0.5%. The summary of dose deviations is illustrated in Fig. 4(a) and 4(b).

**Figure 5:**
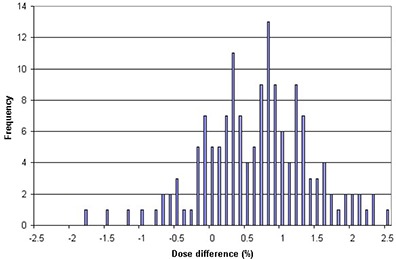
Frequency of dose deviations in normal organs in all transferred plans.

Dose analyses in organs at risk were also performed to supplement the analyses of target doses. They were analyzed as one pool because it is unreliable to analyze the dose deviation differences between the two transfer directions (i.e. from Tomo1 to Tomo2 vs. from Tomo2 to Tomo1) in such dose fall‐off regions. The results show that the average dose deviation is 0.7%±0.5% for RARs and the maximum deviations in absolute values is 2.5%. The summary of dose deviations is illustrated in Fig. 5.

The relationship between patient transfer uncertainties and other uncertainties were analyzed. The latter include the original and transferred plan delivery uncertainties. The resulting Pearson coefficients between PTS‐transfer uncertainties and original plan delivery uncertainties is about 0.07, and between PTS‐transfer uncertainties and transferred plan delivery uncertainties is about −0.1, respectively – indicating that there is no correlation between PTS‐transfer uncertainties and the other two uncertainties. The patient transfer uncertainties have no correlation with dose levels, either. The analyses also show that there is no correlation between the patient transfer uncertainties in target volumes and those in RARs. Resulting Pearson coefficients are summarized in Table 1.

**Table 1:** Summary of Pearson product‐moment correlation coefficients.

*Correlation index of Pearson coefficient*	*Original plan dose delivery uncertainty*	*Transferred plan dose delivery uncertainty*	*Levels of prescribed dose*	*Patient transfer deviations for RARs*
PTS‐transfer deviations for targets	0.07	−0.11	−0.13	0.26
PTS‐transfer deviations for ŔARs	−0.10	0.35	0.11	1.0

Note: Pearson coefficient of ±1 indicates correlation and 0 indicates no correlation. The coefficients listed in this table indicate no or weak correlations.

## IV. DISCUSSION

Our analyses show that there is a slight dose increase with a fraction of a percent in transferred plans compared to original plans (i.e. an increase of 0.8% in target volumes and an increase of 0.7% in normal tissues). These dose deviations reflect the uncertainties induced by the factors that the PTS does not correct. As mentioned in the Introduction section, these factors include machine differences in beam energy, beam profile, junctioning effect, and the application of cutoff thresholding.

Our analyses also show that the dose deviations for plans transferred from Tomo1 to Tomo2 are smaller than those transferred from Tomo2 to Tomo1, which are 0.1% and 1.0%, respectively (see Figs. 4(a) and 4(b)). From the beam model aspect, Tomo1 has lower dose rate than Tomo2. Thus for plans transferred from Tomo1 to Tomo2, leaf open times will be scaled smaller to compensate for higher dose rate of Tomo2, which will cause more small leaf open times under cutoff threshold and lose extra dose in transferred plans. Alternatively, for plans transferred from Tomo2 to Tomo1, leaf open times will be scaled larger, which will bring more leaf open times above the threshold and gain extra dose in the transferred plans. Nevertheless, only a minor number of small leaf times are involved; thus the small leaf influence is not significant.

Patient transfer is a necessary procedure to help patients receive continuous treatments on an alternate machine. It is particularly important for total body irradiation, stereotactic radiosurgery, and head and neck treatment, because the timing of successive fractionations is critical to the outcome of the whole treatment course. If we manually create a backup treatment plan that is identical to the original plan on another tomotherapy treatment planning station, it would take about two hours. However, the PTS takes less than 10 minutes to generate a backup plan.

Other advantages of the PTS are that it does not require user supervision and it provides a batch mode that allows users to submit multiple tasks at one time. The PTS also provides tools that allow dosimetrists or physicists to quantitatively or qualitatively compare a transferred plan with its original plan and evaluate the dose deviations induced by patient transfer.

The beam models of recent tomotherapy machines (after serial number 100) are all tuned to a set of golden beam data, which is similar to the beam‐matching practice done by other vendors,^(4)^ and has the advantage of reducing the commissioning workload and increasing the interchangeability between tomotherapy machines. The patient transfer between such beam‐matched machines would be nearly error‐free because the differences between machines are minimal. Nevertheless, the PTS is indispensable for patient transfer, because a beam‐matched tomotherapy machine owns its unique database and will reject plans generated from other databases.

## V. CONCLUSIONS

Our quantitative evaluation shows that a PTS‐transferred plan is a match to the original plan. The dose deviations between transferred plans and original plans are small and random. With a physician's approval, a transferred plan is acceptable for treatment without the necessity of being revalidated in phantom. Thus far, all of our PTS‐transferred treatment plans have been approved by the attending physicians without further optimization.

## ACKNOWLEDGEMENTS

The authors express their gratitude to Andrea Cox and TomoTherapy Inc. (Madison, WI) for providing us with detailed PTS specifications and for useful discussions.

## References

[1] Mackie TR , Homes T , Swerdloff S , et al. Tomotherapy: a new concept for the delivery of dynamic conformal radiotherapy. Med Phys. 1993;20(6):1709–19. 8309444 10.1118/1.596958

[2] Mackie TR , Balog J , Ruchala K , et al. Tomotherapy. Semin Radiat Oncol. 1999;9(1):108–117. 10.1016/s1053-4296(99)80058-7 10196402

[3] Glantz SA . Primer of Biostatistics. 5th ed. New York (NY): McGraw‐Hill Medical Publishing Division; 2002: 264–67.

[4] Hrbacek J , Depuydt T , Nulens A , Swinnen A , Ven den Heuvel F . Quantitative evaluation of a beam‐matching procedure using one‐dimensional gamma analysis. Med Phys. 2007;34(7):2917–27. 17822000 10.1118/1.2745239

